# Bimetallic Gold–Platinum (AuPt) Nanozymes: Recent Advances in Synthesis and Applications for Food Safety Monitoring

**DOI:** 10.3390/foods14183229

**Published:** 2025-09-17

**Authors:** Shipeng Gao, Xinhao Xu, Xueyun Zheng, Yang Zhang, Xinai Zhang

**Affiliations:** 1 School of Food and Biological Engineering, Jiangsu University, Zhenjiang 212013, China; shipeng.gao@ujs.edu.cn (S.G.);; 2 Key Laboratory of Fermentation Engineering (Ministry of Education), School of Biological Engineering and Food, Hubei University of Technology, Wuhan 430068, China

**Keywords:** gold–platinum nanozymes, bimetallic nanoparticles, enzyme mimics, nanozyme biosensors, food safety

## Abstract

The growing global demand for rapid, sensitive, and cost-effective food safety monitoring has driven the development of nanozyme-based biosensors as alternatives to natural enzyme-based methods. Among various nanozymes, bimetallic gold–platinum (AuPt) nanozymes show superior catalytic performance compared to monometallic and other Au-based bimetallic hybrids. This is due to their synergistic colorimetric, catalytic, geometric, and ensemble properties. This review systematically evaluates AuPt nanozymes in food safety applications, focusing on their synthesis, structural design, and practical uses. Various structural types are highlighted, including plain, magnetic, porous nanomaterial-labeled, and flexible nanomaterial-loaded AuPt hybrids. Key synthesis methods such as seed-mediated growth and one-pot procedures with different reducing agents are summarized. Detection modes covered include colorimetric, electrochemical, and multimodal sensing, demonstrating efficient detection of important food contaminants. Key innovations include core–shell designs for enhanced catalytic activity, new synthesis strategies for improved structural control, and combined detection modes to increase reliability and reduce false positives. Challenges and future opportunities are discussed, such as standardizing synthesis protocols, scaling up production, and integration with advanced sensing platforms. This review aims to accelerate the translation of AuPt nanozyme technology into practical food safety monitoring solutions that improve food security and public health.

## 1. Introduction

Foodborne contaminants represent a substantial global health risk, causing millions of illnesses and deaths annually, with a particular emphasis on children under five years of age [1,2]. Chronic exposure to food hazards is linked to several health issues, including cancer and endocrine disruption [3,4,5]. The increasing internationalization of food trade heightens the risk of contamination, emphasizing the urgent need for rapid and reliable detection technology. Food safety monitoring focuses on a broad range of pollutants, including pathogenic bacteria (e.g., *Salmonella* and *Escherichia coli* O157:H7), mycotoxins (e.g., deoxynivalenol, aflatoxins, and ochratoxin A), antibiotic residues, pesticide residues, heavy metals, and adulterants [6,7,8]. Traditional biosensing methodologies utilize a range of materials and sensing platforms, including enzyme-based biosensors, antibody- or aptamer-functionalized nanomaterials, polymer composites, and other noble metal hybrids [9,10,11]. Although these systems have shown encouraging efficacy in enhancing sensitivity and selectivity, they frequently encounter issues such as inadequate stability, matrix interference, and restricted reusability [12,13].

Noble nanozymes have been methodically examined and refined in biosensing applications due to their unique nanometer scale, adjustable composition and morphology, exceptional catalytic performance, and outstanding stability, enabling their extensive use in biosensing applications [14,15,16,17,18,19]. Gold nanoparticles (Au NPs) are notable biosensing nanozymes due to their biocompatibility, chemical inertness, tunable optical characteristics, and validated synthesis methods [20]. Nonetheless, monometallic Au NPs possess intrinsic constraints that hinder their efficacy in food safety applications. These Au NP nanozymes exhibit moderate catalytic activity relative to normal enzymes, requiring extended reaction durations or extreme conditions to provide adequate analytical sensitivity [21,22]. To mitigate this constraint, bimetallic nanozymes, which demonstrate enhanced catalytic efficacy and adaptability relative to their monometallic equivalents, have been proposed as an alternative.

Among the diverse Au-based bimetallic combinations, gold–platinum (AuPt) nanozymes have surfaced as notably attractive prospects [23]. AuPt nanozymes alleviate the instability of Au-Ag nanozymes due to silver’s susceptibility to oxidative corrosion conditions, while simultaneously enhancing nanozyme activity and broadening the spectrum of enzymatic functions beyond the restricted peroxidase-like activity associated with Au-palladium (Pd) nanozymes [24,25]. Moreover, they address the surface fouling, inadequate stability and biocompatibility of Au-Cu nanozymes, attributed to copper’s tendency for oxidation and leaching in physiological environments, while enhancing the inferior catalytic efficacy and potential toxicity of Au-Ni components [26,27]. In AuPt nanozymes, Pt offers extremely active catalytic sites for H_2_O_2_ activation and substrate oxidation, whilst the gold component enhances Pt’s electronic structure through electronic effects, hence improving catalytic activity [28,29,30]. Moreover, the loading of an ultrathin Pt shell on the Au core can prevent the Pt component from aggregation, thereby remarkably improving the catalytic performance of AuPt nanozymes [21,31]. Significantly, AuPt nanozymes demonstrate many enzyme-like functions in addition to peroxidase activity, hence greatly expanding their use in food safety detection [32,33]. Additionally, AuPt nanozymes exhibit remarkable biocompatibility due to the inherent biological inertness of both Au and Pt. The Au core ensures diminished cytotoxicity and reduced immunogenicity, while the Pt shell is stabilized by the Au core, preventing any leaching [34,35,36]. This structure enhances stability and reduces non-specific interactions with biological elements [25,37]. Furthermore, the surface of AuPt nanozymes can be easily modified with biomolecules such as antibodies, aptamers, and DNA probes, hence improving specificity and selectivity in complex matrices [38,39]. The integration of these biomolecules enhances the effectiveness of AuPt nanozymes by enabling precise identification of foodborne hazards, reducing interference from food matrix components, and ensuring reliable, high-sensitivity detection. As a result, bimetallic AuPt nanozymes have emerged as excellent nanoprobes for the sensitive and precise detection of diverse toxins encompassing a broad spectrum in food samples, including biotoxins [40], pesticide residues [41], heavy metal ions [42], foodborne bacterial pathogens [43,44], mycotoxins [45], fungicide [46], and H_2_O_2_ residues [47], with potential uses extending beyond food safety studies.

Although AuPt nanozymes have demonstrated exceptional performance in monitoring various food contaminants, comprehensive reviews focusing specifically on the advances of AuPt nanozymes in biosensing applications remain scarce [23]. Existing reviews tend to either broadly highlight bimetallic nanozymes across general biosensing applications [19,48,49] or focus on Au-derived bimetallic nanoparticles in diverse sensing scenarios [50,51], without providing dedicated coverage of AuPt nanozymes. A review that systematically includes the recent advances and applications of AuPt nanozymes in food safety monitoring is highly desirable.

**Figure 1:**
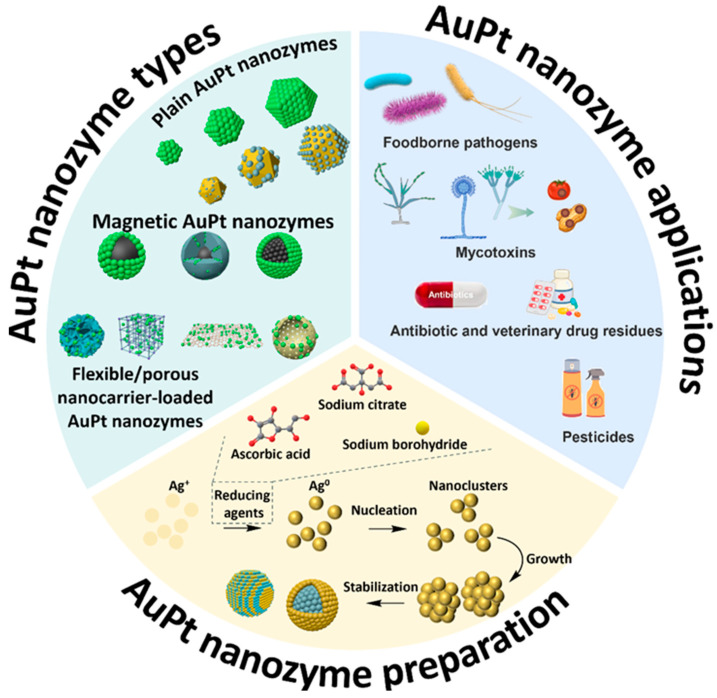
The main context of this review.

This review systematically introduces the synthesis, characterization, and applications of AuPt nanozymes in food safety, covering diverse structural configurations (plain, magnetic, porous nanomaterial-loaded, and labeled on flexible nanomaterial), synthesis methodologies (seed-mediated growth and one-pot approaches), and reducing agent selection (ascorbic acid, sodium citrate, and sodium borohydride) (Figure 1). The review analyzes various detection modes including colorimetric, electrochemical, and multimodal sensing strategies, and their applications in detecting critical food contaminants. Finally, perspectives on overcoming current challenges to achieve commercial viability and enhanced biosensing performance is provided, aiming to accelerate the translation of AuPt nanozyme technology into practical food safety monitoring solutions. Continued endeavors to create superior AuPt nanozymes designed for particular food safety issues are progressing, placing these hybrids at the front of next-generation biosensing technologies.

## 2. Preparation Protocol of AuPt Nanozymes

### 2.1. Type of AuPt Nanozymes

The catalytic activity, stability, and application versatility of AuPt nanozymes are significantly influenced by their structural configuration and composite architecture [52,53,54]. This review categorizes AuPt nanozymes into four specific types according to their material integration strategies. These structural modifications affect the accessibility of active sites and electron transport kinetics while also changing the nanozyme’s adaptability to diverse food matrices.

#### 2.1.1. Plain AuPt Nanozymes

Plain AuPt nanozymes, generally fabricated as core–shell or alloy-type nanoparticles without additional carriers, are the most fundamental and well investigated form of Au–Pt-based nanozymes. Their catalytic activity is profoundly affected by the atomic ratio of Au to Pt and the resulting nanostructure morphology [45]. By modifying the Au:Pt ratio, the density of catalytically active Pt surface atoms, the degree of electronic interaction between the two metals, and the exposure of specific crystalline facets can be regulated, all of which significantly influence catalytic activity [55]. Reduced Au:Pt ratios frequently provide Pt-enriched surfaces that demonstrate improved catalytic activity, while elevated Au levels can augment biocompatibility and electronic conductivity. Moreover, alterations in synthetic methodologies enable the formation of various morphologies, including nanospheres, nanodendrites, nanoflowers, or nanostars [56,57,58,59,60,61]. Structural modifications impact surface area, accessibility of active sites, and electron transfer routes, hence influencing enzymatic kinetics. Thus, plain AuPt nanozymes provide a versatile platform for catalytic improvement and large-scale production, serving as a benchmark for comparison with more complex hybrid nanozyme systems.

The size of Au NP templates is an important factor in controlling the catalytic characteristics of resultant AuPt nanozymes. Three Au NPs of varying sizes, namely G3, G4, and G5, with dimensions of 22, 28, and 34 nm, respectively, were utilized as templates for the synthesis of the AuPt nanozymes, G5Pt, G4Pt, and G3Pt. The average dimensions of the platinum-coated G5Pt, G4Pt, and G3Pt nanoparticles increased by around 5–10 nm, yielding sizes of 39.1, 28.3, and 25.5 nm, respectively. The unmodified Au NPs exhibited a red hue, while the Pt-modified nanoparticles presented a bluish-brown tint. Moreover, the AuPt NPs had a broader absorption spectrum than the pure Au NPs, which primarily absorbed in the 500–600 nm range. The color of the G5 nanoparticles in the 200-fold diluted mixture significantly contrasted with that of the G5Pt nanoparticles, indicating their superior color generation capabilities relative to the uncoated Au NPs [62].

Regulating the ratios of Au:Pt in preparing AuPt nanozymes is also beneficial in enhancing their enzyme-like characteristic, offering superior nanozyme options for biosensing purposes. Au_2_Pt nanozymes exhibit improved efficacy in mediating and accelerating the catalytic reaction process, and the electron density of the Pt atom in Au_2_Pt nanozymes markedly increased compared to that of the Au atom due to the bimetallic doped structure, in contrast to Pt and Au nanoparticles. This feature significantly enhanced the Michaelis constant (*K*_m_) and maximum reaction rate (*V*_max_) of Au_2_Pt nanozymes, yielding *K*_m_ and *V*_max_ values of 0.044 mM and 19.37 × 10^−8^ M s^−1^ for TMB as the substrate, and 6.12 mM and 21.3 × 10^−8^ M s^−1^ for H_2_O_2_ as the substrate [28]. Similarly, utilizing Au NPs (diameter 20.0  ±  2.6 nm) as seeds and adjusting the concentration of Pt^4+^ (20–2000 μM), the resulting Au@Pt nanozymes displayed varying diameters (24–55 nm) and surface areas. Under optimal conditions, Au@Pt NPs exhibited an urchin-shaped morphology, resulting in a 70-fold increase in peroxidase-mimicking activity (specific activity 0.06–4.4 U mg^−1^) and a 30-fold decrease in limit of detection (LOD) through the catalytic activity of Au@Pt, illustrating the effectiveness and applicability in altering the composition of Au@Pt nanozymes [43]. Similarly, an increase in Pt concentration resulted in the gradual formation of a Pt shell on the surface of Au NPs, accompanied by a reduction in absorption peaks. The Pt/Au ratio affects the morphology and catalytic performance of Au@Pt nanozymes, with 24 nm Au@Pt nanozymes exhibiting optimal catalytic activity at a 2:1 (Pt/Au) ratio [63]. The peroxidase-like catalytic activity of Au_x_Pt_y_ NPs were proven markedly exceeded that of Au@Pt and Pt@Au nanoparticles. By adjusting the ratios of Au and Pt atoms (1:9, 2:8, 3:7, 4:6, 5:5, 6:4, 7:3, 8:2, 9:1) in the synthesis of Au@Pt nanozymes, Au_0.4_Pt_0.6_ NPs demonstrated the highest catalytic activity, exhibiting *K*_m_ (2.02 × 10^−3^ M) and *V*_max_ (6.14 × 10^−7^) values that significantly surpassed those of other Au_x_Pt_y_ nanoparticles, along with a strong affinity for H_2_O_2_ [45]. The amount of Pt in Au@Pt nanozymes affects their enzyme-mimicking characteristics and their Raman signal [55]. Au@Pt nanoparticles with 2.5% Pt (Au@Pt_2.5%_) exhibited a satisfactory peroxidase-like feature and produced the most pronounced Raman signal within 2 min, due to the markedly improved catalytic oxidation of TMB substrates, which was facilitated by the Pt coating and the robust electric field maintained by the Au core for surface-enhanced Raman scattering (SERS).

#### 2.1.2. Magnetic AuPt Nanozymes

The integration of Fe_3_O_4_ nanoparticles into AuPt nanozyme systems to prepare magnetic AuPt nanozymes is beneficial in providing functional and operational advantages, particularly within complex food matrices. Firstly, Fe_3_O_4_, being a magnetic substance, enables the rapid and efficient extraction of nanozymes from heterogeneous materials by external magnetic fields, significantly reducing background interference from proteins, lipids, or colored compounds [64,65]. This magnetic responsiveness improves target pre-enrichment and nanozyme recovery while simultaneously enhancing analytical precision by mitigating matrix effects, a notable challenge in practical food safety applications [66,67]. Besides its physical function, Fe_3_O_4_ also fulfills a catalytic role. It demonstrates intrinsic peroxidase-like activity [68], which can enhance the catalytic efficacy of AuPt to optimize overall reaction kinetics. This synergistic effect may arise from improved electron transport, complementary active sites, or cascade-like amplification between the Fe_3_O_4_ core and the AuPt shell. Feng et al. established that the inherent peroxidase-like activity of Fe_3_O_4_ NPs, AuPt NPs, and their combinations adheres to the following order: Fe_3_O_4_@Au–Pt > Fe_3_O_4_ NPs > Au NPs > Fe_3_O_4_–Au, underscoring the critical contribution of the Fe_3_O_4_ core in amplifying catalytic efficacy [69]. The combination of these characteristics makes magnetic AuPt nanozymes highly attractive for reusable, sensitive, and matrix-tolerant biosensing applications.

In the synthesis of magnetic AuPt nanozymes, AuPt can be deposited in situ or sequentially attached on the magnetic core, resulting in magnetic nanozymes with tunable shape. The prior technique utilizes 3-aminopropyltriethoxysilane or ionic liquid functionalization on the magnetic core, facilitating electrostatic adsorption for the deposition of Au and Pt ions [70,71,72]. In contrast, the pre-synthesized AuPt NPs, with customizable size and morphology, can be precisely regulated in their deposition density on the magnetic core, thereby affecting catalytic activity by altering the deposition layers [73]. Specifically, the employment of TiO_2_ nanotubes as carriers facilitates the sequential deposition of Fe_3_O_4_, Au, and Pt nanoparticles, leading to the effective synthesis of magnetic composites with enhanced peroxidase-like activity. This enhancement arises from the significant surface area of the nanotubes, which efficiently promotes the loading of noble nanoparticles, resulting in increased generation of hot electrons to amplify their catalytic activity to enhance the detection sensitivity in monitoring food contaminants [74].

#### 2.1.3. Porous Nanomaterials with AuPt Deposition

Depositing pre-synthesized or in situ synthesized AuPt nanozymes into the interior surface of porous nanostructures is also beneficial in enhancing the catalytic performance of resultant nanocomposites. Specifically, AuPt nanozymes have been anchored inside the channels of various porous nanocarriers, including dendritic SiO_2_ nanospheres [75], zirconium metal–organic framework (Zr-MOF) [76], porous coordination network (PCN-224) [77], and Cu–MOF [78].

Owing to the highly porous three-dimensional mesh structure, these porous nanostructures enhance the efficient immobilization, diffusion, and stabilization of AuPt nanozymes [79]. This feature enhances the effectiveness of the electrochemical reaction by increasing the electrode’s active surface area and providing additional attachment sites for AuPt nanoparticles [75]. Nanocomposites comprising AuPt nanoparticles and porous nanomaterials demonstrate enhanced durability and catalytic efficacy relative to single-metal nanoparticles due to the synergistic interaction between the metals, offering multiple active sites [29]. Additionally, the encapsulation of AuPt NPs within porous nanomaterials can significantly enhance the sensing signal. The amplification of this signal is affected by various factors, particularly the physical confinement effect, which increases the local concentration of catalytic sites, thereby elevating turnover frequencies per unit volume and intensifying the enzymatic reaction signal [80]. Second, the porous architecture facilitates substrate enrichment via adsorption, leading to the accumulation of substrates near the active nanozyme surface and accelerating reaction kinetics [81]. Third, spatial confinement prevents nanoparticle aggregation and protects the nanozyme structure from denaturation or leaching, thereby enhancing stability and preserving activity, especially in unfavorable matrix conditions [82]. These results underscore the critical role of nanoconfinement engineering in improving the performance of AuPt nanozyme-based biosensors in food safety.

#### 2.1.4. AuPt Nanozyme-Anchored Flexible Nanomaterials

Affixing AuPt nanozymes to flexible nanomaterial substrates significantly enhances their dispersion, stability, and integration into biosensors, thereby facilitating the development of remarkable biosensing systems for food hazard detection. Extensively employed flexible nanomaterials—specifically graphene oxide (GO) [83], reduced GO (rGO) [84], Ti_3_C_2_ nanosheets [85], Ti_3_C_2_T_x_ MXenes [42,86], MoS_2_ nanosheets [87], and carbon nanotubes (CNTs) [88,89]—provide significant surface areas, exceptional electrical conductivity, and various functional groups for the efficient immobilization of AuPt nanozymes through electrostatic interactions or covalent bonding. These versatile carriers serve multiple purposes: they prevent nanozyme aggregation, improve electron transport during catalytic activities to enhance the enzyme-like property of nanocomposites, and enable loading abundant nanozymes to result in a superior catalytic activity. Among those selections, GO and rGO offer oxygen-containing functional groups that enhance nanozyme loading and increase hydrophilicity, making them suitable for aqueous food samples [83,90]. Ti_3_C_2_ MXene, distinguished by its surface functional groups and low valence state titanium species, exhibits reducing capabilities that enhance its function as a reductant and support in the synthesis of Au@Pt- Ti_3_C_2_ nanocomposite [86]. This material is distinguished by its configurable surface moieties and improved redox activity, facilitating dual-mode signal production that includes both electrochemical and colorimetric outputs [91,92,93]. CNTs have exceptional flexibility and conductivity, rendering them ideal for functionalizing electrode surfaces to prepare electrochemical biosensors [94,95]. The integration of catalytic amplification from AuPt with the structural benefits of these flexible nanomaterials yields hybrid platforms that exhibit superior sensitivity, rapid response, and exceptional compatibility with miniaturized or portable sensing formats.

### 2.2. Synthesis Methods

#### 2.2.1. Seed-Growth Methods

**Figure 2:**
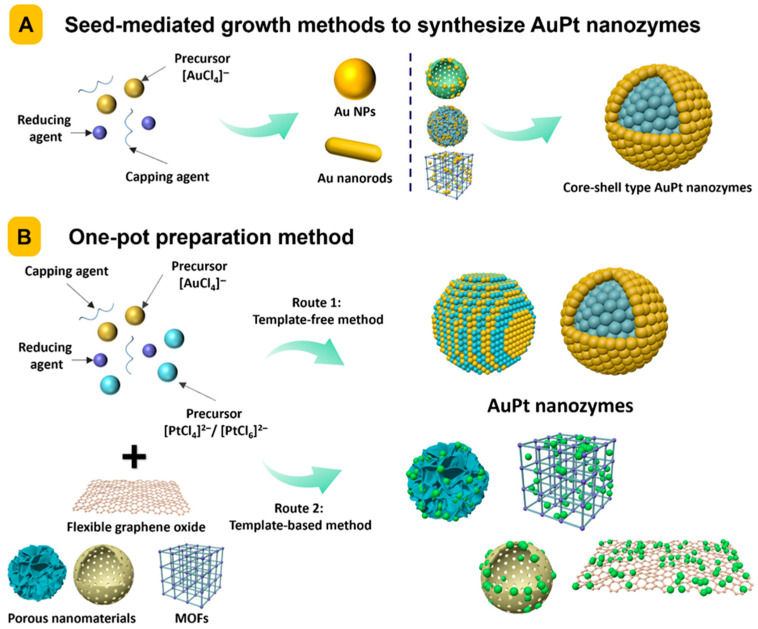
Preparation protocols of AuPt nanozymes.

Seed-mediated growth is a prevalent method for synthesizing AuPt nanozymes, commonly utilizing Au NPs as nucleation centers owing to their specified morphology, superior colloidal stability, and modifiable surface chemistry (Figure 2) [96]. The seed-mediated growth approach typically involves the reduction of Pt precursors (often H_2_PtCl_6_ or K_2_PtCl_4_) in the presence of pre-synthesized Au NPs under controlled pH and temperature settings. The motivation for Pt shell formation stems from the beneficial lattice compatibility between Au and Pt, which diminishes interfacial strain and promotes epitaxial growth. Diverse AuPt nanostructures (e.g., core–shell, branching, or dendritic forms) with adjustable enzyme-mimicking activities can be accurately manufactured by modifying parameters such as Pt precursor concentration, reductant type and concentration, and reaction kinetics [97].

The seed-mediated growth method offers significant advantages in the fabrication of AuPt nanozymes, particularly for morphology regulation, reproducibility, and catalytic performance [61,98,99]. This method enables the accurate control of Pt deposition on prepared Au seeds by separating the nucleation and growth phases, hence allowing the systematic modification of particle size, shape, composition, and surface structure [100]. This control is crucial for improving nanozyme activity, as the catalytic properties of AuPt complexes are profoundly affected by exposed crystal facets, surface roughness, and the distribution of Pt domains [30,101,102]. Moreover, this approach facilitates superior monodispersity and homogeneity in substantial batches, essential for analytical applications requiring reproducible signal output. It mitigates the uncontrolled aggregation commonly observed in one-pot syntheses and provides a versatile framework for the fabrication of anisotropic or hybrid nanostructures (e.g., star-shaped, branching, or core–shell) by only altering the seed geometry or growth conditions [57,103,104]. The seed-mediated process is an effective and scalable approach to fabricate high-performance AuPt nanozymes [105].

Alongside spherical Au NPs, gold nanorods (AuNRs) have recently been investigated as anisotropic seeds to facilitate the epitaxial development of Pt domains, yielding AuPt nanozymes with elongated structures and facet-dependent catalytic characteristics [57,106,107]. The deposition of Pt on AuNR seeds exhibits preferred growth patterns dictated by the crystallographic orientation of specific facet planes. This facet-selective deposition can be employed to generate morphologically diverse AuPt nanostructures, including dumbbell-like, dog-bone, or uniformly coated rod-shaped architectures, each exhibiting distinct catalytic properties [108,109]. These nanorod-based systems not only enhance the structural variety of AuPt nanozymes but also provide potential improvements in catalytic activity and signal amplification owing to enhanced surface area and localized surface plasmon effects [24,39,110].

Recent breakthroughs have explored composite templates that integrate Au NPs with supporting matrices, enhancing structural stability and multifunctional properties beyond typical gold nanostructures. For this target, dendritic silica nanospheres/Au NP [75], Ag@Au-framed nanodisks [111], MIL-100(Fe)/Au NP [112], and Au/Ag NPs [113] have been utilized as templates to enable the controllable growth of Pt NPs on the Au nanostructures, effectively expanding the protocols of AuPt preparation.

#### 2.2.2. One-Pot Synthesis

Compared to the sequential process of seed-mediated growth, one-pot preparation methods offer efficient preparation routes that enable the simultaneous reduction and assembly of Au and Pt precursors into bimetallic AuPt nanozymes [85]. These technologies provide significant advantages in synthetic simplicity, time efficiency, and scalability, making them particularly attractive for commercial purposes. The one-pot methodology encompasses two primary strategies: template-free synthesis and template-assisted techniques.

The template-free one-pot method is the most direct approach for synthesizing AuPt nanozymes, depending on the simultaneous co-reduction of Au and Pt precursors under meticulously regulated conditions [114]. This process often entails the amalgamation of HAuCl_4_ and H_2_PtCl_6_ or K_2_PtCl_4_ in an aqueous solution, succeeded by the incorporation of reducing agents. The efficacy of this method hinges on the tunable control of reduction kinetics. The formation mechanism in template-free systems entails intricate nucleation and growth processes, wherein the initial emergence of Au or Pt nuclei is succeeded by heterogeneous nucleation and alloy synthesis. The ultimate morphology and compositional distribution can be affected by variables such as the Au:Pt precursor ratio, pH, temperature, reduction rate, and the use of reducing agents [21,28,39]. However, a major issue in template-free protocol is attaining a uniform size distribution and regulated morphology, as the lack of structure-directing templates can result in substantial size variations and aberrant forms [24,115]. This constraint can be partially mitigated using kinetic control measures, such as employing mild reducing agents, regulating addition rates, and implementing temperature regulation [29].

Template-assisted one-pot approaches integrate the efficacy of concurrent reduction with the structural regulation afforded by supporting matrices. MOFs, benefiting from their well-defined porous structures, tunable surface chemistry, and ability to confine nanoparticle growth within their cavities, have been utilized as templates for one-pot AuPt nanozyme synthesis [116,117,118]. Specifically, PCN-224 [77], ZIF-67 [29], and Zr-MOF [76] have been utilized. Alternatively, GO has been chosen as a template for the one-pot synthesis of AuPt nanozyme-doped nanocomposites, utilizing its superior dispersion and stabilization of AuPt NPs via π-π interactions and the prevention of nanoparticle aggregation through spatial separation of functional groups [84].

Importantly, the efficacy of one-step self-reduction techniques is fundamentally reliant on the availability of suitable surfactants and polymeric stabilizers, which fulfill several vital roles such as particle size regulation, morphological guidance, and colloidal stability [56,119]. Pluronic F127 functions as a non-ionic block copolymer surfactant, essential for regulating nanoparticle development and inhibiting aggregation [120]. The amphiphilic characteristics of its poly(ethylene oxide)-poly(propylene oxide)-poly(ethylene oxide) (PEO-PPO-PEO) structure facilitate micelle production, which templates nanoparticle synthesis while offering steric stabilization [78,121,122,123]. The polymer’s capacity to coordinate with metal surfaces facilitates the regulation of particle size and morphology during the reduction process [89,124,125]. Cetyltrimethylammonium bromide (CTAB) acts as a cationic surfactant that significantly affects nanoparticle morphology by preferentially binding to particular crystallographic facets [126]. In AuPt synthesis, CTAB can facilitate anisotropic development by preferentially attaching to specific crystal facets, thus regulating the ultimate particle morphology [87]. Polyvinylpyrrolidone (PVP) functions as a polymeric stabilizer, offering steric and electrostatic stabilization via its pyrrolidone functional groups [127,128,129]. The capacity of PVP to interact with metallic surfaces while preserving excellent water solubility renders it an optimal stabilizer for the creation of bimetallic AuPt NPs [130]. The coordination strength of the polymer can be adjusted by selecting the molecular weight, facilitating the optimization of stability and surface accessibility.

### 2.3. Reducing Agents

The choice of suitable reducing agents is a crucial factor in the controlled synthesis of AuPt nanozymes, since these agents directly affect nucleation kinetics, growth processes, particle morphology, and the final structural properties of the bimetallic system [131]. The reducing agent not only enables the transformation of metal precursors from ionic to metallic states but also significantly influences the reduction sequence, which is especially important in bimetallic systems where several metals possess unique reduction potentials [132,133]. A thorough comprehension of the mechanistic elements and selection criteria for diverse reducing agents is crucial for attaining optimal AuPt nanozyme characteristics (Table 1).

The reduction mechanism of AuPt nanozymes generally occurs in several specific stages [28,29,152,153]: (1) initial electron transfer from the reducing agent to the metal precursor; (2) formation of metal atoms and subsequent nucleation; (3) growth via ongoing reduction and atomic addition; and (4) possible surface reactions, including alloy formation or core–shell restructuring. The interaction among these processes dictates whether the resultant product displays core–shell architecture, alloy formation, or separated domain structures.

#### 2.3.1. Ascorbic Acid

Due to the mild and pH-responsive reduction ability, ascorbic acid (vitamin C) has emerged as one of the most commonly employed reducing agents in the preparation of AuPt nanozymes, owing to its gentle reduction characteristics, biocompatibility, and pH-dependent reduction kinetics [43,59,72,97,145]. The reducing capacity of ascorbic acid is closely associated with its molecular structure, which has two adjacent hydroxyl groups that can be oxidized to produce dehydroascorbic acid, simultaneously releasing electrons for the reduction of metal ions [154,155].

The pH-dependent characteristics of ascorbic acid reduction present both a benefit and a significant factor in synthesis design. Under acidic conditions (pH < 4), ascorbic acid demonstrates limited reducing capacity, facilitating a gradual reduction that promotes consistent nucleation and development [155]. As pH approaches neutral and alkaline values, the reducing strength markedly increases due to the deprotonation of hydroxyl groups, hence accelerating reduction rates [156]. The pH sensitivity facilitates temporal regulation of the reduction process, permitting sequential reduction techniques in which pH modification governs the timing and degree of Au vs. Pt reduction.

In the manufacture of AuPt nanozymes, the mild characteristics of ascorbic acid render it especially appropriate for seed-mediated growth techniques, wherein regulated Pt deposition onto pre-existing gold seeds is sought [60,62,63,127,143]. As conducted by Pham et al., a low concentration of the Pt^2+^ precursor and ascorbic acid were incrementally introduced to the SiO_2_@Au seeds at 5-min intervals to facilitate precise control over the size of the Pt NPs. These mildly reducing conditions permit enhanced regulation of the Pt layer’s growth, as the reaction progresses at a significantly slower rate compared to strongly reducing conditions [47]. The comparatively gradual reduction kinetics facilitate homogeneous shell formation while reducing undesirable secondary nucleation.

#### 2.3.2. Sodium Citrate

Sodium citrate holds a distinctive role among reducing agents in preparing AuPt nanozymes since it functions as both a reducing and a stabilizing agent. The citrate ion possesses many carboxylate groups that can align with metal surfaces, offering electrostatic and steric stability while also acting as an electron donor for the reduction of metal ions [46,69,148,149,150]. The dual functionality of sodium citrate renders it very helpful in one-pot synthesis methods that need simultaneous reduction and stabilization [45,114,136].

The reduction mechanism of citrate entails the oxidation of the hydroxyl group next to the carboxylate moiety, generally taking place at elevated temperatures (80–120 °C) [129,136]. The reducing power of citrate is moderate, situated between the mild characteristics of ascorbic acid and the vigorous reduction of borohydride. This intermediate strength facilitates regulated nucleation while supplying adequate driving force for the thorough reduction of both Au and Pt precursors [157].

The chelating characteristics of citrate in bimetallic AuPt synthesis can affect the reduction sequence by generating complexes with metal ions of varying stabilities. The enhanced complexation with Pt ions relative to Au ions can vary the effective reduction potentials, potentially changing the inherent thermodynamic preference for Au reduction [131]. The complexation phenomenon, along with temperature-dependent reduction kinetics, provides precise control over the creation mechanism and ultimate structure of AuPt nanozymes [158].

Citrate adsorption on the surfaces of resulting nanoparticles stabilizes them, preventing aggregation during synthesis while ensuring adequate surface accessibility for ongoing growth [159]. The resultant citrate-capped nanozymes frequently demonstrate superior colloidal stability and can be easily functionalized via ligand exchange processes for targeted applications. More interestingly, the stabilizing effect of citrate differs from that of other stabilizers, including thiol end-capping compounds. Studies indicate that citrate can establish a monolayer covering at low concentrations, but thiol end-capping agents necessitate greater concentrations to attain a comparable result [160].

#### 2.3.3. NaBH_4_

Sodium borohydride (NaBH_4_) is another often utilized reducing agent in nanozyme synthesis, effectively reducing Au and Pt precursors swiftly under ambient circumstances [70,71]. The hydride ion acts as a strong electron donor, facilitating reduction with hydrogen evolution as a byproduct: BH_4_^–^ + 8OH^–^ → BO_2_^–^ + 6H_2_O + 8e^–^. This reaction offers a sustained supply of electrons with significant reducing potential, facilitating swift and thorough reduction of metal precursors [58,75,137].

The potency and speed of borohydride reduction offer both benefits and difficulties in the synthesis of AuPt nanozymes [161]. Rapid kinetics may induce burst nucleation, yielding elevated nucleation density and diminutive particle sizes, which can enhance surface area and catalytic efficacy. Nonetheless, the same swift kinetics may induce kinetic entrapment of non-equilibrium structures, potentially culminating in alloy formation instead of the thermodynamically favored core–shell design.

In sequential reduction procedures, the application of excess borohydride may result in the concurrent reduction of both metal precursors, necessitating meticulous stoichiometric regulation to attain the intended architectural results [162]. The addition methodology is crucial, utilizing dropwise addition or controlled release procedures to regulate reduction rates and oversee nucleation and development processes. Alkaline circumstances commonly linked to borohydride reduction, resulting from hydrolysis processes, might affect the stability and shape of developing nanoparticles. The elevated pH environment can influence the surface charge of particles and the ionization state of stabilizing agents, necessitating the consideration of pH buffering or post-synthesis pH modification to attain optimal stability and catalytic efficacy [163].

The selection of reducing agent significantly influences both the synthesis results and the ultimate characteristics and efficacy of AuPt nanozymes in food safety applications. Therefore, comprehensively comparing and evaluating the characteristics of those reducing agents are crucial in obtaining desired AuPt nanozymes. In comparison between those three agents, ascorbic acid is ideal for regulated, sequential reduction procedures where moderate conditions and biocompatibility are essential. The pH-dependent activity facilitates facile temporal control strategies, rendering it especially appropriates for seed-mediated development methods aimed at precisely specified core–shell architectures. Sodium citrate presents benefits in one-pot synthesis techniques where simultaneous reduction and stabilization are required, since its modest reducing capability ensures a balance between control and efficiency. NaBH_4_ is favored for rapid and thorough reduction, especially in high-throughput synthesis or when minimal particle sizes are necessary for optimal catalytic activity. Nonetheless, its application necessitates meticulous kinetic regulation to prevent undesired alloy formation or aggregation.

## 3. Different Detection Modes of AuPt Nanozyme-Based Biosensing Methods

### 3.1. Colorimetric Mode

Nanozyme-mediated colorimetric sensing has emerged as a prominent technique for detecting food hazards owing to its simplicity, speed, cost-effectiveness, and ease of use [164,165]. A core design strategy in these bimetallic AuPt nanozyme-assisted colorimetric sensing systems is to harness the peroxidase or oxidase-like activity of nanozymes to catalyze chromogenic reactions, typically involving substrates like as 3,3′,5,5′-tetramethylbenzidine (TMB), 2,2′-Azinobis [3-ethylbenzothiazoline-6-sulfonic acid]-diammonium salt (ABTS), or o-phenylenediamine dihydrochloride (OPD), thereby producing visually detectable signals for target analyte identification [56,58,142]. AuPt nanozyme-driven colorimetric techniques can be configured in either competitive or sandwich formats, depending on the size characteristics of the analytes (e.g., small molecules, proteins, pathogens, or mycotoxins) [134,151,166]. Competitive formats are typically suitable for small molecules or haptens, where the analyte competes with labeled analogs for limited binding sites [151]. In contrast, sandwich-type assays are more appropriate for larger targets, such as proteins or bacterial pathogens, as dual recognition (capture and detection) enhances both selectivity and signal amplification [120]. The modularity in assay design enhances flexibility and adaptability for different food contaminants and allows integration with portable or smartphone-based devices, hence facilitating on-site, user-friendly food safety monitoring systems.

Lateral flow assays (LFAs), or immunochromatographic assays, utilize AuPt nanozymes as signal tracers, are distinguished by their simplicity, rapid and real-time analysis, visual recognition, high specificity, and low cost [167]. Driven by those advantages, AuPt nanozyme-based colorimetric LFAs have been utilized to monitor *Staphylococcus aureus* (*S. aureus*) [138], ofloxacin [97], mycotoxins [104], okadaic acid [40], 3-phenoxybenzoic acid [63], pesticide and antibiotic residues [73,83,143,144], diaminochlorotriazine [59], bacterial pathogens [43,62], displaying promising applicability and versatility. Interestingly, by alternating the corresponding antibodies on the AuPt nanozymes, multiplexing detection of several analytes can be realized, enabling the high throughput screening of different food hazards [125]. More advantageously, the remarkable thermostability of nanozymes offers substantial benefits over conventional enzymes in minimizing background signals when utilized as signal probes [14]. This work indicates that the heightened endogenous peroxidase activity in maize extract generally produces a substantial background signal, thereby reducing both specificity and sensitivity. The application of Joule heating in a portable battery-operated device rapidly raised the temperature of the LFA test strips to 75–80 °C, therefore denaturing the natural enzymatic activity while maintaining the properties of the nanozyme, in accordance with the standard LFA protocol. As a result, this Au@Pt nanozyme-based lateral flow assay significantly reduced background noise and improved the limit of detection by a factor of 3.5 compared to the experiment performed without heating. Interestingly, aptamers have been alternatively utilized in preparing those LFA methods [143,144], expanding the options of bioreceptors and offering additional affordability advantages.

Alternatively, AuPt nanozyme-assisted colorimetric sensor arrays have exhibited exceptional capability in simultaneously detecting and distinguishing various foodborne risks [114,123]. Sensor arrays utilize many sensing units with diverse catalytic reactions to provide multi-dimensional response patterns, referred to as “chemical fingerprints,” unlike single-channel colorimetric tests [168]. Au_2_Pt nanozymes, demonstrating remarkable peroxidase-like activity (*K*_m_ of 0.044 mM and *V*_max_ of 19.37 × 10^−8^ M s^−1^ for TMB), were utilized as signal probes to create a colorimetric sensor array for evaluating the total antioxidant capacity of food. Based on the differing reduction capabilities of antioxidants in suppressing the generation of oxidized TMB. This sensor array possessed a detection threshold of under 0.2 μM and demonstrated ability in effectively assessing total antioxidant capacity in milk, green tea, and orange juice samples [28]. AuPt-assisted colorimetric sensor arrays provide multiple benefits, such as the adjustable and rational design of nanozymes with diverse yet complementary reactivities towards various analytes through the modulation of their composition and morphology, dependable signal generation and readout in intricate food matrices, and the facilitation of non-specific, pattern-recognition detection of structurally similar analytes that conventional sensors cannot selectively identify [169].

### 3.2. Electrochemical Mode

Leveraging the inherent electrocatalytic properties and improved electrical conductivity of Au and Pt components, AuPt nanozymes demonstrate considerable synergistic effects that markedly improve charge transfer efficiency and catalytic kinetics at the electrode–electrolyte interface [88]. The properties of AuPt nanostructures make them exceptionally efficient electroconductive nanoprobes in electrochemical biosensors, improving signal amplification and increasing detection sensitivity [170,171]. AuPt nanozymes enhance redox reactions and reduce overpotential barriers, hence facilitating more sensitive and stable electrochemical transduction, even at low analyte concentrations [172,173]. Thus far, many food contaminants have been precisely identified utilizing AuPt-assisted electrochemical devices, encompassing small-molecule toxins such as bisphenol A [88], aflatoxin B1 (AFB_1_) [130], fumonisin B1 (FB_1_) [124], ochratoxin A (OTA) [76], H_2_O_2_ [135], and furosemide [78]. These examples underscore the considerable usability and performance benefits of AuPt nanozymes in electrochemical sensing devices intended for food safety monitoring.

To enhance the sensitivity of AuPt nanozyme-assisted electrochemical sensors, various nanomaterials have been integrated to function as signal nanotracers or surface modifiers. The “confinement effect” of zeolitic imidazolate framework-8 (ZIF-8) was employed for the incorporation of Au NPs, resulting in Au@ZIF-8 nanocomposites used for electrode deposition on multi-walled CNTs (MWCNTs), while toluidine blue (TB)-loaded hollow spherical cerium dioxide (CeO_2_)/AuPt nanohybrids acted as signal tracers for electrochemical sensing [130]. Similarly, AuPt NPs/Zr-MOF functioned as an electrode modification material, providing several active sites and improving electron transfer rates, leading to a signal amplification of 1.47 times [76]. Alternatively, Au NPs@MXenes were synthesized to act as a sensing substrate on the electrode surface, while Au@Pt nanocrystals, exhibiting excellent peroxidase-like activity, served as nanocatalysts to facilitate the H_2_O_2_-mediated TMB reaction, thereby producing a robust differential pulse voltammetry (DPV) signal for the enhancement of a competitive aptasensor for FB_1_ [124]. Also in a competitive format, the electrochemical signal can be obtained from molecular beacons. Specifically, the presence of analytes will cause the detachment of cDNA from the aptamer/cDNA complex to form a stable double-stranded structure with methylene blue (MB)-modified capture probes, thereby triggering Exo III to cut the MB-DNA/cDNA, resulting in a sharp drop in the MB signal [61].

Alternatively, AuPt NPs have been modified on the electrode surface in preparing those electrochemical sensors. AuPt NPs were utilized as electrocatalysts on screen-printed carbon electrodes (SPCE) owing to their superior electrocatalytic reduction efficiency for H_2_O_2_. The resultant non-enzymatic electrochemical sensor facilitated the sensitive detection of H_2_O_2_ in contaminated milk, attaining a sensitivity of 155.5 µA·mM^−1^ cm^−2^ and an LOD of 2.5 µM. The sensor demonstrated commendable reproducibility, with a relative standard deviation (RSD) below 4%, and maintained consistent performance over four months. Moreover, the facile integration with a portable electrochemical analyzer-simulator enhances the sensor’s practical utility for on-site verification of H_2_O_2_ adulteration in raw cow milk samples [135]. A composite of AuPtPd trimetallic nanoparticle-functionalized MWCNTs and chitosan-modified glassy carbon electrode (GCE) was proposed for detecting bisphenol A in food. The catalytic characteristics of nanocomposites were utilized to oxidize bisphenol A substrates, and the resultant changes in the electrochemical DPV signal were observed for analyte measurement [88].

### 3.3. Other Detection Methods

Magnetic relaxation switching (MRS) biosensors, which leverage the state fluctuations of magnetic nanoparticles (MNPs) to change the transverse relaxation time (Δ*T*_2_) of nearby water molecules, enabling the preparation of highly sensitive biosensors for the detection of numerous food hazards [174]. MRS has been used to monitor foodborne bacterial illnesses owing to its simplicity, rapidity, exceptional signal-to-noise ratio, and appropriateness for on-site detection [175,176]. To address the insufficient sensitivity, restricted linear range, and low resistance to food interferents of the Fe^2+^/Fe^3+^ conversion method in the development of an MRS biosensor, the conversion of Mn(VII) to Mn(II) was utilized to offer an extended electron relaxation time and a strong magnetic signal, thereby improving the signal-to-noise ratio and detection sensitivity [177]. As explained in an earlier work, Au@Pt nanozymes served as catalysts to decompose H_2_O_2_, and the residual H_2_O_2_, initiated the conversion of Mn(VII)/Mn(II) to cause a signal variation of Δ*T*_2_ signal for the quantification of bacterial pathogens [141].

Photoelectrochemical (PEC) sensing has evolved as a potent analytical method for food safety assessment, attributed to its little background signal, elevated sensitivity, and straightforward equipment [178]. PEC sensors provide the precise identification of many food contaminants by transforming light energy into electrical impulses through photoactive nanoparticles, and the integration of AuPt nanozymes with photoactive substrates can further amplify PEC signals via improved charge separation and catalytic efficacy [179,180]. Mulberry-like Au@Pd@Pt dendritic nanorods served as nanocatalysts to oxidize diaminobenzidine (DAB) to produce insoluble precipitates and thereby reduce the photocurrents, while the presence of AFB_1_ released the suppression effect of precipitates to result in a higher photoelectrochemical signal [107].

An electrochemiluminescence system was introduced employing SnS_2_ quantum dots (QDs) and Cys-AuPt heterogeneous nanorings as an effective coreaction accelerator and luminophore. In this nanocomposite, AuPt nanodonuts exhibit notable electrochemical properties, facilitating the production of supplementary coreactant intermediates in the SnS_2_ QDs/K_2_S_2_O_8_ system, thus significantly amplifying the ECL signal of SnS_2_ QDs with the assistance of L-Cys [111].

### 3.4. Dual and Multiple Modes

The integration of dual or multiple detection modes into a unified nanozyme-based sensing platform has proven to enhance analytical robustness, sensitivity, and adaptability in several food safety applications [181]. Researchers have effectively combined colorimetric, electrochemical, photothermal, and SERS techniques into an integrated system by leveraging the multifunctional attributes of AuPt, which encompass its strong catalytic activity, plasmonic characteristics, and electron transfer proficiency. These hybrid platforms provide complementary signal generation and cross-validation, reducing false positives and negatives while augmenting detection confidence, especially in complex food matrices [182,183].

The bifunctionality of oxTMB molecules, resulting from the AuPt oxidation process, has enabled the advancement of dual-mode approaches employing colorimetric indicators and Raman reporters. Incubating glucose oxidase (GOx) with Au@Pt/Fe-DACDs initiates a cascade reaction, converting glucose into H_2_O_2_, which then acts as a substrate for nanozymes to oxidize TMB, yielding quantifiable and visually identifiable Raman-active oxTMB. This method successfully accomplished the selective detection of glucose in serum, with an LOD of 2.3 μM for the colorimetric sensor and 1.4 μM for SERS sensor [147]. Similarly, the blocking impact of analytes obstructs catalytic sites on the MIP platform, leading to a reduced catalytic reaction of TMB and thus diminishing the UV-visible signal or Raman intensity. A dual signal output mode for saxitoxin detection was established, integrating colorimetric and SERS methods [71]. Similarly, a colorimetric and SERS dual-mode aptasensor was created for the detection of chloramphenicol. This study utilized flower-like Fe_3_O_4_@Au as a magnetic separator and SERS substrate, while Au@Pt nanozyme served as a catalyst to facilitate colorimetric alterations and amplify the Raman signal during the transformation of TMB to oxTMB, thus activating the aptasensor. Concurrently, the presence of chloramphenicol triggers the exponential amplification reaction (EXPAR), producing a significant amount of amplicon DNA that adopts a “Y-shape” configuration, thereby promoting the closeness of Au@Pt nanozyme to Fe Fe_3_O_4_@Au to boost the SERS signal. This methodology exhibited LODs of 9.23 × 10^–9^ M and 4.96 × 10^–13^ M for colorimetric and SERS modes, respectively, and validated its reliability with HPLC in identifying chloramphenicol in milk [129].

Leveraging the exceptional optical density and fluorescence quenching properties of oxTMB molecules, a colorimetric and fluorescence dual-mode enzyme-linked immunosorbent assay (ELISA) biosensor was developed for the detection of imidacloprid. Employing rhodamine 6G dyes as fluorescence indicators, oxTMB diminished the fluorescence signal, achieving LODs of 0.88 μg/L and 1.14 μg/L in colorimetric and fluorescence modes, respectively, surpassing conventional ELISA (1.21 μg/L). Furthermore, this method demonstrated satisfactory applicability for detecting imidacloprid in samples of Chinese cabbage, cucumber, and zucchini [148]. Alternatively, guanosine monophosphate (GMP)-protected Au-Pt nanoclusters with intrinsic fluorescence intensity were served as catalysts and fluorophores for the colorimetric and fluorescence detection of glucose. Utilizing OPD as a substrate, the oxidized fluorescent molecule 2,3-diaminophenazine (DAP) demonstrated fluorescence emission at 560 nm, diminishing the inherent fluorescence intensity of nanozymes and enabling a ratiometric fluorescence detection technique. This dual readout technology achieved LODs of 7 μM and 11 μM for the respective modes, demonstrating notable reliability and recovery rates in spiked serum samples [140].

## 4. Applications of AuPt Nanozymes in Detecting Food Contaminants

### 4.1. Bacterial Pathogens

Foodborne pathogen-related acute and chronic illnesses are global health issues. These pathogenic bacteria can develop in poorly prepared, stored, or handled food matrices. Chicken, eggs, and fresh vegetables contain *Salmonella* spp. (e.g., *Salmonella typhimurium*, *S. typhimurium*), which can cause gastrointestinal and systemic diseases [184]. *L. monocytogenes*, which can tolerate cold temperatures, can cause septicemia, meningitis, and fetal loss in immunocompromised and pregnant persons who eat ready-to-eat meats, dairy, and smoked salmon; undercooked ground beef and leafy greens can cause hemorrhagic colitis and hemolytic uremic syndrome with *Escherichia coli* O157:H7 [185,186]. Due to the low infectious dose of many pathogens and the risk of widespread outbreaks, rapid, sensitive, and precise detection technologies are needed to monitor bacterial contamination throughout the food supply chain to ensure prompt intervention and reduce foodborne disease impacts on public health systems [187,188]. As shown in Table 2, examples of utilizing AuPt nanozymes in determining different food contaminants have been summarized.

**Figure 3:**
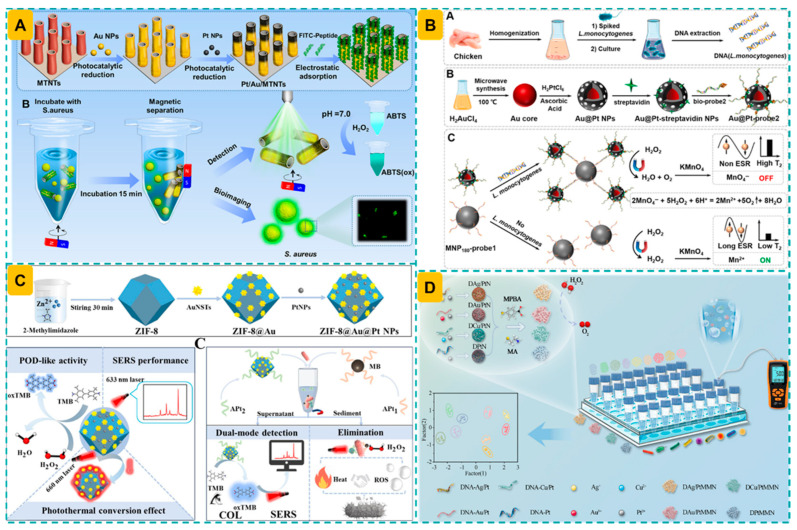
AuPt nanozyme-assisted biosensors for monitoring foodborne bacterial pathogens. (**A**) Pt nanoparticle-decorated Au/TiO_2_ magnetic nanotubes exhibiting remarkable peroxidase-like activity were employed to detect Staphylococcus aureus, utilizing the surface plasmon resonance effect of Au nanoparticles, achieving a detection limit of four cells. Reprint with permission from [74]. (**B**) A magnetic relaxation switching aptasensor mediated by Au@Pt nanozymes was developed for the sensitive detection of Listeria monocytogenes in chicken samples. Reprint with permission from [141]. (**C**) ZIF-8@Au@PtNPs, exhibiting significant peroxidase-like activity, SERS, and photothermal conversion properties, was utilized for the development of a dual-mode detection system and collaborative eradication of Listeria monocytogenes. Reprint with permission from [79]. (**D**) A pressure sensor array based on four functionalized DNA-nanoenzymes was developed for multiplex detection of foodborne pathogens in tap water and raw beef samples. Reprint with permission from [137].

The successive deposition of Au NPs and Pt NPs on TiO_2_ magnetic nanotubes resulted in asymmetric Au/Pt/MTNTs nanocomposites that demonstrated enhanced efficiency in producing hot electrons, hence enhancing the catalytic activity of Au/Pt nanozymes [74]. Fluorescein isothiocyanate (FITC)-labeled peptide probes were adsorbed onto Au/Pt/MTNTs, while the presence of *S. aureus* induced the detachment of probes, restoring the peroxidase-like activity of nanozymes. This resulted in an LOD of four cells, along with commendable selectivity and practicality in detecting *S. aureus* in milk and juice samples (Figure 3A).

To enhance the detection sensitivity of AuPt nanozyme-derived nanozymes, an alternative method was proposed. Utilizing Au@Pt nanozymes as signal nanoprobes and amplifiers, an MRS DNA sensor was established for the sensitive detection of *L. monocytogenes* [141]. Herein, DNA of *L. monocytogenes* that was extracted from the chicken samples served as a linker to conjugate DNA-decorated MNPs and Au@Pt nanozymes, exhibiting a lower Δ*T*_2_ signal. Based on the reverse relationship between *L. monocytogenes* concentrations and Δ*T*_2_ signal, an LOD of 30 CFU/mL was obtained, which was 33-folds more sensitive than that of ELISA. Moreover, this MRS method exhibited highly comparable results with qPCR, while addressing the disadvantages of sample pretreatment and tedious procedure (Figure 3B).

To improve the result reliability, biosensors with multiple readout modes was designed. A colorimetric and SERS dual-mode detection of *L. monocytogenes* was established, facilitated by the multifunctionality of ZIF-8@Au@Pt nanoparticles, which exhibit peroxidase-like activity, SERS characteristics, and photothermal conversion capability [79]. An aptasensor comprising aptamer_1_-conjugated magnetic beads and aptamer_2_-attached ZIF-8@Au@Pt nanozymes was utilized to produce oxTMB, exhibiting a unique SERS fingerprint facilitated by magnetic separation. This technique, using the bifunctionality of ZIF-8@Au@Pt nanozymes, achieved LODs of 7 and 5 CFU/mL for two modes, demonstrating durability after 15 days of storage and application in identifying bacteria in milk, pork, and lettuce samples. The remarkable photothermal conversion efficiency of nanozymes (η = 71.72%) facilitated nearly complete eradication of *L. monocytogenes* within 2 min of near-infrared irradiation, hence preventing potential secondary contamination (Figure 3C).

To address the challenge of multiplexed analyte detection, a nanozyme-enhanced pressure sensor array was developed for the simultaneous identification of foodborne pathogens, extending beyond the detection and monitoring of individual pathogen types [137]. This study utilized four nanoenzymes (Ag/Pt, Au/Pt, Cu/Pt, Pt) exhibiting catalase-like activity as nanocatalysts for the creation of a pressure sensor array. The unique interaction between bacteria and functionalized 4-mercaptophenylboronic acid (MPBA) and β-mercaptoethylamine (MEA) on the nanozyme surface generated varied pressure response patterns by catalyzing H_2_O_2_ to O_2_, hence altering the pressure within a sealed tube. Nine bacterial pathogens were detected and isolated by chemometric approaches to evaluate pressure responses, employing a portable pressure manometer that showed high sensitivity and accuracy in detecting artificially contaminated samples (100% in tap water and 91.7% in raw beef) (Figure 3D).

### 4.2. Mycotoxins

Mycotoxins, secondary compounds from fungi, influence food safety and health. Grain, nut, fruit, and processed foods contain these chemicals, especially under high humidity and poor storage [189]. The International Agency for Research on Cancer classified *Aspergillus* spp. aflatoxins (e.g., AFB_1_) as Group I carcinogens in corn, peanuts, and dried fruits [190]. Nephrotoxic OTA in grapes, coffee, and grains may cause cancer; *Fusarium* fumonisins (e.g., FB_1_) in maize cause esophageal cancer and neural tube abnormalities; other mycotoxins that cause endocrine and gastrointestinal issues include zearalenone (estrogenic, frequently found in corn and wheat) and patulin (found in decaying apples and fruit juices) [191,192,193]. Due to food safety authorities’ low limitations and mycotoxins’ wide distribution in food matrices, regulatory enforcement requires rapid, sensitive, and accurate detection technologies to decrease exposure and protect human health [194,195].

**Figure 4:**
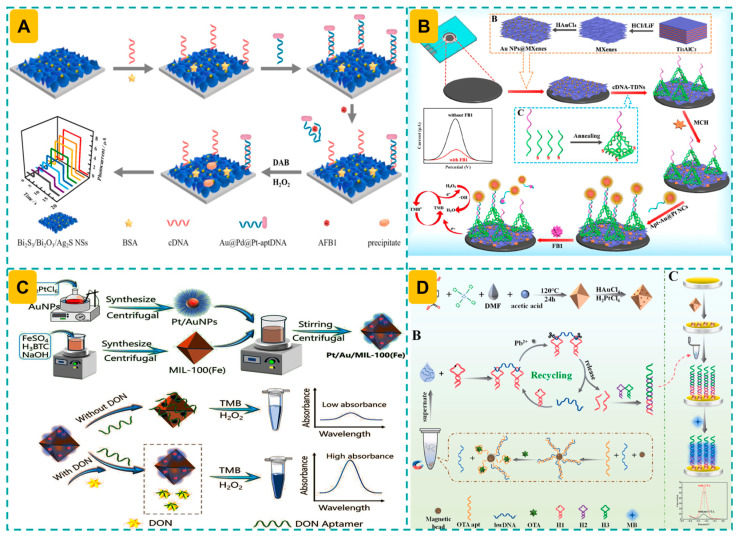
AuPt nanozyme-assisted biosensors for monitoring mycotoxins. (**A**) A photoelectrochemistry aptasensor based on ternary Bi_2_S_3_/Bi_2_O_3_/Ag_2_S heterostructured nanosheets was constructed for the ultrasensitive detection of AFB_1_, assisted by Au@Pd@Pt dendritic nanorod nanozymes. Reprint with permission from [107]. (**B**) Au NPs@MXenes with excellent conductivity were used as the sensing substrate and modified onto the electrode for electrochemical detection of fumonisin B1, using Au@Pt nanocrystals as signal tracers. Reprint with permission from [124]. (**C**) A colorimetric aptasensor based on Pt/Au nanoparticles functionalized metal–organic frameworks (Pt/Au/MIL-100(Fe)) was developed for the detection of deoxynivalenol in spiked wheat and maize flour samples. Reprint with permission from [112]. (**D**) A label-free homogeneous electrochemical sensor for ochratoxin A detection was constructed using AuPt NPs/Zr-MOF as the electrode-modified material, coupled with a cascade amplification strategy involving a π-structure bipedal DNA walker-triggered HCR. Reprint with permission from [76].

A competitive “signal-on” PEC aptasensor was developed for the sensitive detection of AFB_1_ [107]. This study presents dual II-scheme sheet-like Bi_2_S_3_/Bi_2_O_3_/Ag_2_S heterostructures that exhibit efficient separation of electron-hole (e^−^-h^+^) pairs, notable stability, and improved photoactivity, utilized for signal amplification in electrodes. The introduction of AFB_1_ analytes will induce the dissociation of Au@Pd@Pt nanozymes from the Bi_2_S_3_/Bi_2_O_3_/Ag_2_S substrates, leading to an amplified signal. This technology achieved a broad linear range of 0.5 pg/mL to 100 ng/mL and a low LOD of 0.09 pg/mL, based on the signal recovery efficiency of nanozymes. This technique can identify spiked analytes in peanut milk samples, with recovery rates between 97.0% and 102.9%, with an RSD ranging from 3.05% to 4.18% (Figure 4A).

Targeting the portable detection of mycotoxins, a paper substrate-based electrochemical aptasensor was developed for the detection of FB_1_ [124]. Conductive Au NPs@MXenes were utilized as a sensing substrate on the electrode to promote the subsequent attachment of tetrahedral DNA nanostructures (TDNs), whereas Au@Pt nanocrystals served as signal nanoprobes. An elevated concentration of FB_1_ will initiate the strand displacement process, resulting in a diminished nanozyme loading density, thereby creating a linear range from 50 fg/mL to 100 ng/mL, with an LOD of 21 fg/mL. This technique was appropriate for measuring analytes in corn and wheat samples, with a recovery rate of 96.4% to 104.8% and an RSD between 2.48% and 3.72% (Figure 4B).

Aiming to enhance the detection sensitivity of AuPt nanozyme-assisted biosensors, different methods have been constructed. A colorimetric aptasensor was created for the detection of deoxynivalenol (DON), utilizing the aptamer-modulated nanozymatic activity of Pt/Au nanoparticles functionalized with metal–organic frameworks (Pt/Au/MIL-100(Fe)). In the presence of DON, the aptamer dissociated from the Pt/Au/MIL-100(Fe) surface, reinstating the peroxidase-like activity of the nanozyme and producing a measurable colorimetric signal. The sensor demonstrated an advantageous linear detection range (50–5000 ng/mL) and a low LOD of 44.14 ng/mL, with findings exhibiting strong agreement with those acquired using HPLC in detecting DON in real samples. Furthermore, the nanozyme exhibited remarkable storage durability, preserving catalytic efficacy with an RSD < 3% after 21 days at 4 °C (Figure 4C). Integrating AuPt NPs/Zr-MOF, DNAzyme-driven bipedal DNA walker and HCR, a label-free electrochemical sensor for OTA detection was developed [76]. This research utilized AuPt NPs/Zr-MOF for electrode modification, improving the density of active sites and conductivity, resulting in a 1.47-fold signal enhancement. Furthermore, the elongated double chain produced by the HCR mechanism facilitated the efficient integration of MB molecules for subsequent signal amplification. This method, enhanced by the synergistic effects of various signal amplification techniques, demonstrated notable stability (maintaining over 82.6% of the initial current after 21 days of storage at 4 °C), reproducibility (intra- and inter-batch RSDs of 1.4% and 2.1%, respectively), and applicability to real samples (recoveries ranging from 93.6% to 108.6% for corn flour, coffee powder, and black tea) (Figure 4D).

### 4.3. Heavy Metal Ions

Heavy metal ions in the food chain can harm people, even at low amounts. Lead ions (Pb^2+^), cadmium ions (Cd^2+^), mercury ions (Hg^2+^), and arsenic ions (As^3+^) are detected in food matrices due to industrial pollution, agricultural runoff, and inadequate waste management [196,197]. Leafy vegetables and root crops absorb Pb and Cd from contaminated soil, while seafood, especially large predatory fish, is a major source of methylmercury, a neurotoxin that causes irreversible brain damage [198]. Group I carcinogen inorganic As is rapidly absorbed in flooded paddy fields [199]. Due to their non-biodegradability and bioaccumulation, heavy metal ions in food must be identified rapidly, sensitively, and correctly to prevent their health threat to humans [200,201].

By incorporating AuPt into the pores of dendritic silicon nanospheres featuring central-radial porous structures, the resultant AuPt@DSN nanocomposite exhibits remarkable peroxidase-like activity and dispersibility [75]. The presence of Hg^2+^ specifically and swiftly suppressed the catalytic activity of nanozymes, resulting in diminished colorimetric signal intensity. According to this concept, the sensor exhibited an LOD of 8.58 pM and a linear range from 0.1 nM to 10 μM. This sensor facilitates the determination of Hg^2+^ within 20 min and demonstrates efficacy in detecting Hg^2+^ in tap water, Hengqing lake water, and polluted water samples. Likewise, the inhibitory influence of Ag^+^ on the Au@Pt nanozyme-mediated decomposition of H_2_O_2_ under moderately acidic circumstances, this LPSR spectroscopy method enabled the selective detection of Ag^+^ across a dynamic concentration range of 0.5 to 1000 μM, with an LOD as low as 500 nM [106]. The approach exhibited remarkable recovery efficiency for Ag^+^ in both tap and spring water samples, underscoring its superior selectivity, sufficient sensitivity, and exceptional stability.

To satisfy the need of multiplexing detection of several heavy metal ions, a sensor array consisting of three signal recognition components (AuPt@Fe-N-C, AuPt@N-C, and Fe-N-C) was developed for the rapid, precise, and high-throughput identification of Hg^2+^, Pb^2+^, Co^2+^, Cr^6+^, and Fe^3+^ [114]. Heavy metal ions can be recognized and measured based on their suppression efficacy in affecting the catalytic color synthesis of the chromogenic substrate (TMB). The colorimetric sensor array and smartphone-based RGB mode can detect concentrations as low as 0.5 μM, including binary and ternary mixes, in less than five minutes. The sensor array accurately evaluated 10 μM quantities of heavy metals in seawater and salmon samples, demonstrating great portability and sensitivity.

### 4.4. Antibiotic and Veterinary Drug Residues

Due to their harmful effects and role in the global antimicrobial resistance epidemic, antibiotic and veterinary medicine residues in food threaten public health and food safety [202]. These residues originate from widespread use of antibiotics, antiparasitics, and growth-promoting medicines in cattle, aquaculture, and food production [203,204]. Poor withdrawal durations or dosing may leave drug molecules in meat, milk, eggs, fish, and other animal products. Common residue types include tetracyclines, sulfonamides, β-lactams, macrolides, quinolones, and antiparasitic medicines like ivermectin and levamisole. Small levels of these residues can cause hypersensitivity, gut flora change, hepatotoxicity, and carcinogenicity. Food with subtherapeutic antibiotics may preferentially push microorganisms, increasing multidrug-resistant diseases. Regulatory bodies’ tight maximum residue limits (MRLs) and food matrices’ complex composition require fast, sensitive, and reliable detection technologies for ensuring consumer health [205,206].

**Figure 5:**
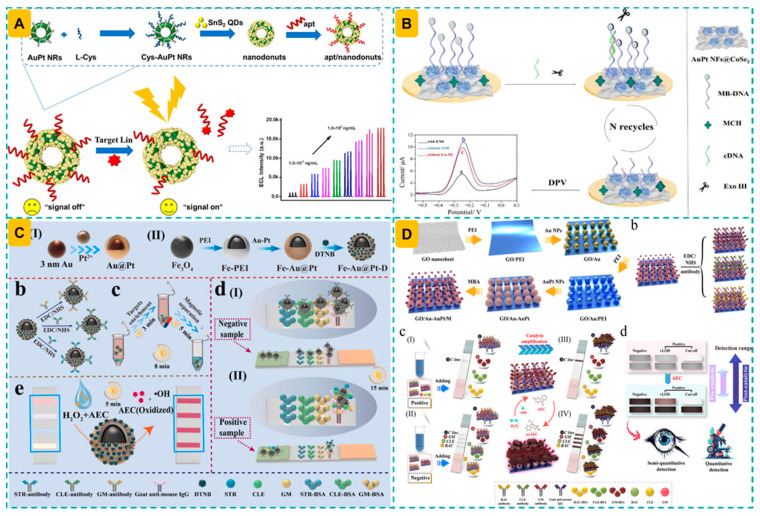
AuPt nanozyme-assisted biosensors for monitoring antibiotic and pesticide residues. (**A**) A label-free electrochemiluminescence aptasensor for luminophore detection was developed based on SnS_2_ quantum dots/Cys-AuPt heterogeneous nanorings. Reprint with permission from [111]. (**B**) A sensitive electrochemical sensor for enrofloxacin detection in milk was constructed by integrating an Exo III-assisted signal amplification strategy with Au@Pt NFs/CoSe_2_ nanosheets as the substrate material. Reprint with permission from [61]. (**C**) A core–satellite-structured magnetic nanozyme (Fe_3_O_4_–Au@Pt)-based multiplex LFA was constructed for simultaneous and ultrasensitive detection of gentamicin, streptomycin, and clenbuterol within 30 min. Reprint with permission from [73]. (**D**) A 3D sheet-like GO/Au–AuPt nanozyme-based competitive immunochromatography assay was constructed for simultaneous monitoring of gentamicin, clenbuterol, and ractopamine in pork, chicken, lake water, and river water samples. Reprint with permission from [83].

A label-free electrochemiluminescence aptasensor was developed for the sensitive detection of lincomycin using SnS_2_ QDs/Cys-AuPt NRs as signal probes [111]. This method demonstrated an LOD of 0.7 fg/mL and a linear range spanning from 1 fg/mL to 0.1 pg/mL. No notable change in ECL intensity was noticed after 10 cycles of continuous potential scanning, with the RSD of ECL intensity measured at 2.61%, demonstrating remarkable stability and reproducibility. The identification of analytes in milk produced recovery rates between 98.25% and 104.8%, whereas the RSD of the aptasensor varied from 3.9% to 6.2% (Figure 5A).

Improving the number of AuPt nanozymes on the nanostructure, the resulting nanoprobes is prone to exhibit an enhanced detection sensitivity. Employing polyethyleneimine as a linker, a substantial quantity of Au@Pt nanoflowers was affixed to the lamellar CoSe_2_ nanosheets to enhance the electrode’s functionality, consequently improving conductivity and facilitating the creation of an aptasensor for enrofloxacin detection [61]. The presence of analytes that compete with pre-formed aptamer/cDNA facilitates the release of cDNA, hence initiating the Exo III-mediated signal amplification process. The LOD was obtained at 1.59 fg/mL, exhibiting satisfactory repeatability (RSD = 1.24%) and applicability to real samples, as evidenced by analyte recovery rates in milk ranging from 95.7% to 104.2%, with an RSD range of 1.71% to 3.43% (Figure 5B).

Multiplexing LFAs are crucial in simultaneously revealing the co-existence of many food hazards, providing higher portability and rapidity [207]. Fe_3_O_4_-Au@Pt-type core–satellite-structured nanozymes were utilized to prepare a colorimetric LFA for monitoring three common veterinary drugs, namely, gentamicin (GM), streptomycin (STR), and clenbuterol (CLE) [73]. Herein, the electrostatic adhesion of abundant Au@Pt nanoparticles onto the Fe_3_O_4_ core resulted in nanocomposites exhibiting enhanced peroxidase-like activity and magnetic separation capabilities. This LFA, enhanced by the dual-signal amplification from magnetic enrichment and catalytic enhancement (which expanded the linear range by 27–85 times post-catalytic reaction), achieved multiplex detection of STR, CLE, and GM within 30 min, with LODs of 10.1, 6.3, and 1.1 pg/mL, respectively. Its usefulness in identifying drug residues in honey, milk, and pig samples has also been validated (Figure 5C). Alternatively, GO functioned as a nanocarrier to enable the successive deposition of 20 nm Au NPs and 5 nm AuPt NPs for the fabrication of three-dimensional (3D) sheet-like nanotags [83]. This approach achieved a 3.4-fold enhancement in sensitivity and an 83.3-fold increase in detection range, facilitated by the remarkable peroxidase-like activity of flexible nanozymes through catalytically amplified signals. This method showed significant performance in identifying drug residues (GM, CLE, and ractopamine) in several authentic samples (e.g., pork, chicken, lake water, and river water), demonstrating exceptional precision, stability, and specificity (Figure 5D).

### 4.5. Pesticide Residues

Pesticide residues in food can cause endocrine disruption, neurotoxicity, and cancer [208,209]. Due to agricultural pesticide use, fruits, vegetables, and cereals often contain these residues [210,211]. To ensure food safety and reduce health risks, pesticide residues must be identified and measured. Compared to traditional methods, nanozyme-assisted biosensors may detect pesticide residues quickly, cheaply, and portably using nanozymes’ catalytic properties [212].

AuPt alloy nanoparticles decorated on CeO_2_ nanorods (CeO_2_@AuPt NRs) were utilized for detecting malathion, a representative organophosphate pesticide [54]. In this approach, oxTMB, produced through the oxidation of CeO_2_@AuPt NRs, was inhibited by ascorbic acid following the hydrolysis of L-ascorbic acid-2-phosphate in the presence of acid phosphatase, resulting in a weakened color signal. The presence of malathion restored the colorimetric signal, enabling detection with an LOD of 0.085 U/L.

Alternatively, an enzyme-free bio-barcode immunoassay was developed for detecting parathion, utilizing the catalytic ability of Au@Pt nanozymes [41]. In this method, a mixture of nanoelements—including antibody and ssDNA-labeled Au NPs, parathion ovalbumin (OVA)-hapten-modified MNP probes, and complementary DNA (cDNA)-conjugated Au@Pt probes—was employed. Parathion competed with MNP probes to bind antibodies on the Au NP probes, releasing Au@Pt nanozymes that initiated a catalytic reaction to generate a colorimetric signal. This method achieved an LOD of 2.13 ng/kg, significantly improving the sensitivity compared to traditional peroxidase-driven methods (LOD = 20.82 ng/kg). Moreover, this biosensor showed excellent correlation with results obtained from liquid chromatography–tandem mass spectrometry (LC–MS/MS) for detecting spiked samples of rice, pear, apple, and cabbage.

AuPt nanozyme-assisted dual-mode detection methods have also been proposed to enhance detection reliability. For instance, carbendazim fungicide competed with cDNA-labeled Fe_3_O_4_ nanoparticles to bind aptamers on the Au@Pt surface, resulting in varying amounts of Au@Pt nanozymes in the supernatant and magnetic precipitate. This allowed for both naked-eye visualization of the dark blue color and absorbance quantification using a multifunctional microplate reader. The dual readout method showed excellent agreement with LC–MS/MS for monitoring carbendazim, with correlation coefficients of 0.9339 and 0.9321 for leek and rice, respectively [46]. Additionally, a colorimetric and fluorescence dual-mode assay was developed for detecting imidacloprid, utilizing the fluorescence quenching ability of oxTMB molecules [148]. In this system, imidacloprid and OVA-hapten on a 96-well plate competed for antibody binding on Au@Pt. An increase in imidacloprid concentration reduced the binding of Au@Pt-Ab to the OVA-hapten, leading to corresponding changes in signal intensity. The colorimetric signal exhibited an inverse relationship with imidacloprid concentration, while fluorescence showed a positive linear correlation, resulting in LODs of 0.88 μg/L and 1.14 μg/L for colorimetric and fluorescence modes, respectively.

**Figure 6:**
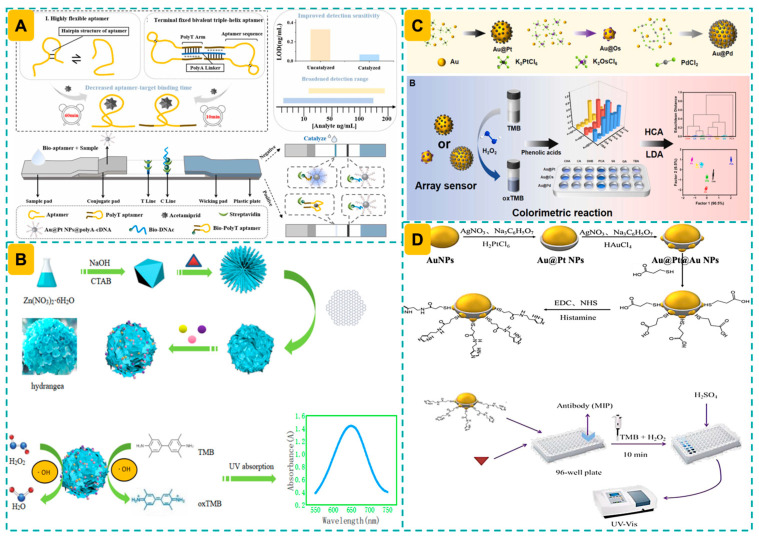
(**A**) An aptamer-based lateral flow assay for acetamiprid detection was constructed using Au@Pt nanozymes to catalyze TMB color development, with signal readout enabled by a smartphone-based device. Reprint with permission from [144]. (**B**) A colorimetric sensor based on hydrangea-like AuPtRu/ZnO-rGO was developed for sensitive detection of H_2_O_2_ in milk samples. Reprint with permission from [84]. (**C**) A nanozyme-based colorimetric sensor array incorporating Au@Pt, Au@Os, and Au@Pd was constructed for the detection of seven phenolic acids, with machine learning-assisted pattern recognition enabling accurate discrimination. Reprint with permission from [123]. (**D**) A sensitive biomimetic ELISA for histamine detection was developed using Au@Pt@Au composite nanozymes. Reprint with permission from [150].

To improve the portability of pesticide detection, Mao et al. developed a lateral flow test for acetamiprid detection using a bivalent triple helix aptamer with two 10 nt arms, resulting in a sixfold higher binding rate than the original aptamer [144]. Au@Pt NPs@polyA-cDNA hybridizes with bio-polyT aptamer in the absence of acetamiprid, and streptavidin (SA) immobilized on the T line captures the complex. Aggregated Au@Pt NPs catalyzed the chromogenic substrate, increasing Apt-LFA strip signal strength. Acetamiprid prevents its binding to the bio-polyT aptamer, interrupting Au@Pt NPs@polyA-cDNA hybridization and reducing test line signal. This colorimetric approach demonstrated an LOD of 0.068 ng/mL and a linear range that is five times lower and four times broader than that of the Au NP-based LFA (Figure 6A).

### 4.6. Food Adulterants and Other Food Hazards

H_2_O_2_ is frequently utilized as a food adulterant, especially in dairy products and fruit juices, to improve whiteness or prolong shelf life [213]. Nonetheless, its presence in food presents considerable health hazards, including oxidative stress, cellular damage, and possible carcinogenic effects [214]. Consequently, the identification of H_2_O_2_ is essential for safeguarding food safety and preserving public health. The peroxidase-like activity of AuPt nanozymes can be easily employed to develop a colorimetric biosensor for the sensitive detection of H_2_O_2_, characterized by simplicity and versatility [47,135]. Zinc oxide nanoflowers and AuPtRu NPs co-modified reduced GO demonstrated remarkable peroxidase-like activity, enabling label-free colorimetric detection of H_2_O_2_ under mildly acidic circumstances. The synthesized nanocomposites exhibited an improved catalytic rate (*V*_max_ = 6.16 × 10^–8^ M/s) and affinity (*K*_m_ = 0.02) for H_2_O_2_, enabling colorimetric detection of H_2_O_2_ within the range of 5–1000 μM, with an LOD of 3.0 μM, and recovery rates ranging from 93.0% to 101.7% in milk samples [84] (Figure 6B).

Cysteine, an antioxidant and dough-conditioner, is another typical food adulterant that frequently added to baked foods. The Au@Pt-decorated MoS_2_ nanosheet exhibited remarkable catalytic efficacy for the oxidation of TMB, whereas the presence of cysteine impeded the catalytic reaction, thereby diminishing the production of oxTMB. This phenomenon facilitated the selective quantification of cysteine in buffer and supplement tablets with high sensitivity (0.5 μM), stability, and reproducibility [87].

A colorimetric sensor array was created utilizing Au@Pt, Au@Os, and Au@Pd nanozymes, which demonstrated distinct peroxidase-like activities, aimed at the screening and differentiation of various phenolic acids [123]. The selective inhibitory effects of bimetallic nanozymes on catalytic activities were employed to investigate the distinct colorimetric signals of chlorogenic acid (CHA), protocatechuic acid (PCA), syringic acid (SA), gallic acid (GA), 2,3,4-trihydroxybenzoic acid (TBA), caffeic acid (CA), and 2,5-dihydroxybenzoic acid (DHB) through principal component analysis (PCA) and linear discriminant analysis (LDA) at concentrations ranging from 0.005 to 0.01 mM, both individually and in combination. The LODs for DHB and TBA were established at 1.7 μM and 1.3 μM, respectively, and the ability to distinguish between various phenolic acids in real water samples was utilized to confirm their practical applicability (Figure 6C).

A competitive biomimetic ELISA utilizing Au@Pt@Au nanoparticles as signal tracers was proposed for the sensitive detection of histamine in a 96-well plate format [150]. MIPs served as biomimetic antibodies, providing simplicity, cost-effectiveness, and reusability. Under optimal conditions, this method achieved an LOD of 0.069 mg/L and a sensitivity of 7.20 mg/L. Furthermore, spiked histamine concentrations in yellow rice wine and liqueur samples were accurately quantified, yielding recoveries between 84.28% and 108.82%, with results corroborated by the HPLC method (Figure 6D).

## 5. Perspective

Bimetallic AuPt nanozymes have emerged as promising candidates for food safety research owing to their distinctive structural characteristics, remarkable catalytic efficiency, and compatibility with various sensing systems. Their applications encompass different targets and enable multiple signal output modalities. Nonetheless, several significant challenges persist, such as limited structural controllability during synthesis, insufficient catalytic activity, and the lack of standardized performance evaluation methods. Future research should concentrate on resolving these challenges.

### 5.1. Developing and Optimizing Protocols of Synthesizing AuPt Nanozymes with Higher Controllability and Greenness

One-pot synthesis methods for AuPt nanozymes are frequently adapted due to their simplicity and scalability; yet, they often demonstrate insufficient control over nanoparticle morphology, surface structure, and shell uniformity, all of which are critical for catalytic activity and reproducibility. The seed-mediated synthesis of prepared Au NPs as cores enables meticulous control over the thickness, content, and spatial arrangement of the Pt shell, resulting in extremely homogeneous core–shell nanostructures with varied catalytic characteristics. However, seed preparation, surface functionalization, and controlled Pt deposition are generally required, consequently increasing operational complexity and reducing synthetic throughput. Therefore, there is a growing focus on sustainable and regulated synthesis methods to tackle these challenges. The synthesis of AuPt nanozymes using microfluidic chip technology enables continuous, automated, and precisely regulated manufacturing under mild reaction conditions [31]. Laminar flow and microscale mixing in microfluidic reactors facilitate accurate nucleation and growth kinetics, improving batch consistency and reducing reagent usage [215]. Flow-based systems reduce waste and enable parallelization, making them sustainable chemical processes. The fabrication of programmable and eco-friendly AuPt nanozymes for biosensing applications can be expedited by incorporating real-time monitoring and feedback control in microfluidic synthesis systems [216,217]. To achieve this objective, Artificial Intelligence (AI) and Machine Learning techniques can provide accurate synthetic regulation parameters and enhanced insights into the predictive understanding of how structural factors affect catalytic efficacy [218]. Machine learning algorithms can discern subtle correlations, improve synthesis conditions, and predict optimal nanostructure designs with minimal experimental input by analyzing high-dimensional datasets of reaction parameters (such as precursor ratios, temperature, and duration), nanoparticle morphology, and catalytic activity metrics [219,220]. AI-driven models and microfluidic synthesis platforms may enable real-time, adaptive management of reaction parameters informed by in-line characterization feedback, facilitating fully autonomous nanozyme production with exceptional repeatability and environmental efficiency. This demonstrates significant potential for the systematic development and scaling of next-generation AuPt nanozymes for biosensing applications as nanozyme databases expand [221,222].

### 5.2. Integration of Other Nanomaterials for Preparing Superior AuPt Nanozymes

Although AuPt nanozymes have demonstrated promising catalytic activity, integrating additional nanomaterials with AuPt has been frequently employed to enhance the nanozyme characteristics of nanocomposites [223]. Hybridization with flexible nanostructures (e.g., MoS_2_, GO or MWCNTs) significantly enhances electron transfer efficiency and colloidal stability to facilitate the electrochemical detection, while simultaneously boosting substrate accessibility through high surface area interfaces [83,224]. Likewise, the incorporation of AuPt with MOFs enhances selectivity driven by confinement and cascade catalytic activity, owing to their tunable porosity and functionalized coordination environments [225]. The incorporation of metal oxides (e.g., CeO_2_ nanorods) provides redox-active sites that augment the catalytic properties of the nanozyme beyond peroxidase-like activity, facilitating multi-enzyme mimicry [73]. Specifically, besides their inherent peroxidase-like activity, Fe_3_O_4_ NPs have magnetic responsiveness, facilitating rapid separation and concentration of target analytes from viscous or particulate-laden environments and substantially reducing background noise and matrix effects caused by proteins, lipids, or pigments commonly present in food products [226,227,228]. These multifunctional nanozyme composites exhibit enhanced catalytic kinetics and enable integration into advanced biosensing formats. In the future, these material-engineering approaches are expected to be essential in creating next-generation AuPt-based nanozymes with tailored specificity, improved turnover, and robust performance in complex food matrices [137].

### 5.3. Exploring Au or Pt Alternatives to Expand Nanozyme Diversity and Adaptability

AuPt nanozymes exhibit superior catalytic performance and stability; however, the scarcity and high cost of noble metals necessitate the integration or substitution of these elements with more cost-effective transition metals—such as Ag, Cu, Co, or Ni—thereby presenting a feasible strategy for developing next-generation nanozymes that enhance economic viability and catalytic diversity [229,230,231]. Alternative metals can be integrated into bimetallic or alloy structures (e.g., Ag@Pt, Cu–Au, Pt–Co) through systematic design, where their unique electrical and surface properties may not only preserve but often enhance enzyme-like functionalities by modifying the electron density distribution of active sites, thereby enhancing redox processes essential for peroxidase- or oxidase-like activity [232]. These materials engineering strategies provide dual benefits: they significantly lower synthesis costs while enhancing the diversity of available nanozyme functions. The enhanced functional diversity is especially advantageous for creating biosensors specifically designed for various food matrices, where pH, ionic strength, and interfering substances fluctuate considerably [233]. The employment of less expensive metals facilitates scalable production and wider application of nanozyme-based assays in resource-constrained or high-throughput screening environments [234,235,236]. An exhaustive examination of substitutes for Au or Pt is essential for the economical, application-oriented, and sustainable advancement of nanozymes in food safety assessment [237,238]. Moreover, in evaluating and comparing the catalytic efficiency of these nanozyme alternatives, density functional theory (DFT) functions as a comprehensive tool for examining the catalytic reaction mechanism of TMB substrates on the nanozyme surface, facilitating the comparison of the projected density-of-states (DOS) of different nanozymes and clarifying the underlying mechanisms that improve the catalytic performance of specific nanozymes [28]. It helps to clarify how architectural design affects the functional performance of AuPt nanozymes and guides their methodical advancement for practical food safety detecting protocols.

### 5.4. Establishing Multiple Signal Integration Methods with High Portability and Applicability

Meanwhile, the enhancement of device-level integration is essential for translating nanozyme performance into functional analytical platforms [146]. The development of various signal transduction techniques provides redundancy, enhanced sensitivity, and adaptability across diverse detection scenarios [139,148]. Microfluidic chips enable automated, miniaturized, and multiplexed detection with precise fluid management and little reagent consumption, while their adaptation to LFAs permits rapid, instrument-free, and user-friendly on-site testing [239,240,241]. The catalytic amplification properties of AuPt nanozymes can surpass the sensitivity limitations often associated with paper-based or portable assays [99,124,242]. Foreseeing future developments, the incorporation of these nanozyme systems with smartphone-based readout modules, wireless data collection, and sample-to-answer configurations will be crucial for the establishment of authentic field-deployable point-of-care (POC) testing technologies tailored for food safety monitoring [120,243,244]. Moreover, it would be advantageous to utilize emerging bioreceptors (e.g., boronic acid derivates, peptides, and nanobodies) to replace commercially available antibodies to reduce the cost and improve the effectiveness in recognizing and separating analytes from complex food samples [74,245,246,247]. The availability of numerous bioreceptor alternatives enhances flexibility in the development of biosensors, enabling the creation of more cost-effective and portable biosensing systems for food safety [248].

Considering the multiplexing analysis, sensor arrays exhibit significant benefits in the concurrent identification and quantification of several analytes at varying concentrations, using the “fingerprinting” attributes derived from nanozyme-catalyzed products [249]. Rapid, high-throughput differentiation can be accomplished by combining array output with chemometric methods, demonstrating significant affordability, label-free operation, cost-effectiveness, and user-friendly on-site screening [250]. Furthermore, its integration with smartphone imaging or microfluidic technologies to prepare integrated devices may enhance POC diagnostics and commercialization [251,252].

### 5.5. Standardization of AuPt Nanozyme Definition and Activity Assays

Recent advancements in validated testing protocols for nanozyme activity facilitate the standardization of AuPt nanozyme activity evaluation across various production methods and surface morphologies [253]. These advancements accelerate the creation of quantitative, precise, and scalable methodologies for activity evaluation that enhance repeatability, and provide cross-platform comparison [254]. The transition from laboratory-scale synthesis to commercial production requires addressing scalability challenges while maintaining uniform quality and performance of nanozymes [255]. Strategies for process optimization utilizing statistical design of experiments and machine learning algorithms enable the identification of critical process parameters and their optimal ranges for large-scale synthesis [256]. Cost-effectiveness analysis demonstrates the economic viability of producing AuPt nanozymes at commercial sizes, including raw material expenses, processing requirements, and quality control costs relative to traditional detection methods [257]. These initiatives enhance the applicability and reliability of AuPt nanozymes in food safety applications and establish a foundation for their wider adoption in point-of-need testing and regulatory frameworks. The comprehensive standardization and validation efforts promote regulatory approval and commercial adoption, while the development of scalable manufacturing processes ensures that AuPt nanozyme technology can meet the rising global demand for rapid, accurate, and cost-effective food safety monitoring solutions. Furthermore, these advancements facilitate the incorporation of AuPt nanozymes into current food safety frameworks, allowing for effortless implementation by regulatory bodies, food processing enterprises, and global field-testing requirements.

## 6. Conclusions

Due to continuous progress in interdisciplinary research in materials science, nanocatalysis, and device engineering, AuPt nanozymes are set to become vital elements in the future of biosensing systems. Their remarkable catalytic activity, stability, and adaptability, together with the capacity for integration with diverse nanomaterials including magnetic, porous, and flexible substrates, render them very efficient for food safety monitoring. Magnetic AuPt nanozymes have demonstrated significant potential for improving analyte recovery and improved detection sensitivity in intricate food matrices. Furthermore, porous AuPt nanozymes exhibit exceptional efficacy in signal amplification, rendering them optimal for the detection of low-concentration pollutants. Core–shell architectures provide the most substantial enhancements in catalytic performance, while advancements in synthesis techniques, including as seed-mediated growth and one-pot approaches, have enhanced control over size, morphology, and activity. These developments, especially when combined with multi-modal sensing platforms, have facilitated more dependable and economical solutions for food safety monitoring.

Consequently, although all structural kinds and methodologies enhance detection capacities, emerging AuPt nanozymes have produced the most promising outcomes, particularly for real-time and on-site food safety applications. To improve, it is essential to tackle issues including the standardization of synthesis techniques, the scaling of production, and the integration with sophisticated sensing platforms to fully harness the potential of AuPt nanozymes in food safety, thereby enhancing food security and public health globally.

## Figures and Tables

**Table 1:** Preparation methods and used reducing agents in synthesizing AuPt nanozymes.

Preparation Methods		Reducing Agents			
		Ascorbic Acid	Sodium Citrate	NaBH_4_	Other Agents
**One-pot synthesis**	**Template-free**	[28,54,56,78,89,98,99,106,120,121,123,124,125,134,135]	[45,114,136]	[52,58,130,137,138,139]	[140]
	**Template-assisted**	––	––	[76,77,84]	[29,85,86]
**Seed-growth methods**	**Au NPs as seeds**	[16,41,43,44,47,59,60,62,63,72,73,87,88,97,127,128,141,142,143,144,145,146,147]	[46,69,129,148,149,150]	[70,71]	[14,40,53]
	**Au NRs as seeds**	[57,107,122]	––	––	––
	**Other nanomaterials as seeds**	[104,111,112,113]	––	[75]	[151]

**Table 2:** AuPt nanozyme-assisted sensitive monitoring various food contaminants.

Analytes	Au/Pt Nanozymes	Detection Methods	Detection Limit	Real Samples	Ref.
**Bacterial pathogens**					
*Clavibacter michiganensis*	Au@Pt	Lateral flow immunoassay	300 CFU/mL	Potato tuber extract	[43]
*Salmonella typhimurium*	Au@Pt	Colorimetric	12 CFU/mL	Chicken	[44]
*Escherichia coli* O157:H7	Dumbbell Au-Pt	Colorimetric	2 CFU/mL	Tap water and romaine lettuce	[57]
*Staphylococcus aureus*	Au/Pt nanoclusters	Colorimetric	80 CFU/mL	Milk, orange juice and human serum	[58]
*Staphylococcus aureus*	Fe_3_O_4_/TiO_2_ nanotubes/Au NP/Pt NP	Colorimetric	4 cells	Milk and juice	[74]
*Escherichia coli* O157:H7	Sea cucumber-like AuPt/PCN-224	Colorimetric (naked-eye, absorption spectra, and smartphone)	10, 1, and 2 CFU/mL	Lake water, lettuce and milk	[77]
*Listeria monocytogenes*	ZIF-8@Au nanostar@PtNPs	Colorimetric and SERS dual-mode	7 and 5 CFU/mL	Milk, pork, and lettuce	[79]
*Staphylococcus aureus*	Porous Au@Pt	Colorimetric	40 CFU/mL	Milk	[98]
*Escherichia coli* O157:H7	Au@AuPt	Pressure meter (O_2_)	3 CFU/mL	Water and tea	[99]
*Salmonella*	Au@Pt	Colorimetric (microfluidic)	168 CFU/mL	Pork meat	[127]
*Salmonella typhimurium*	Au@Pt	Colorimetric	17 CFU/mL	Chicken meat	[128]
*Salmonella*	Au@PtPd	Colorimetric (Finger-actuated microfluidic chip)	45 CFU/mL	Pork	[134]
Nine Pathogens	DNA-Ag/Pt, DNA-Au/Pt, DNA-Cu/Pt, and DNA-Pt-nanoenzyme	Pressure sensor array	10^2^ and 10^4^ CFU/mL	Tap water and raw beef	[137]
*Staphylococcus aureus*	Ultrasmall AuPtIrRuRh	Lateral flow immunoassay (colorimetric and catalytic colorimetric)	1.5 × 10^3^ and 15 CFU/mL	Milk and orange juice	[138]
*Listeria monocytogenes*	Au@Pt	Magnetic relaxation switching	30 CFU/mL	Chicken	[141]
*Salmonella*	Au@Pt	Colorimetric	56 CFU/mL	Skim milk and ultrapure water	[145]
*Escherichia coli* O157:H7	Au@AuPt	ELISA	100 CFU/mL	Tap water and milk tea	[166]
**Mycotoxins**					
Aflatoxin B1	Au@Pt	Lateral flow immunoassay	4 pg/mL	Corn	[14]
Aflatoxin B1	AuPt@ZIF-67	Flow-injection chemiluminescence immunoassay	0.68 pg/mL	Corn and wheat	[29]
Zearalenone	Au_0.4_Pt_0.6_	Colorimetric	0.6979 ng/mL	Wheat and corn	[45]
Ochratoxin A	AuPt@IL@Fe_3_O_4_	Colorimetric	0.078 ng/mL	Beer and corn	[72]
Ochratoxin A	AuPt NPs/Zr-MOF	Electrochemical	0.525 pg/mL	Corn flour, black tea and coffee powder	[76]
Zearalenone	Pt@Au nanoflower	Lateral flow immunoassay	0.052 ng/mL	Corn	[104]
Aflatoxin B1	Dendritic nanorod-like Au@Pd@Pt	Photoelectrochemical	0.09 pg/mL	Peanut milk	[107]
Deoxynivalenol	Pt/Au/MIL-100(Fe)	Colorimetric	44.14 ng/mL	Wheat and maize flour	[112]
Fumonisin B1	Au@Pt	Electrochemical	21 fg/mL	Corn and wheat	[124]
Deoxynivalenol and zearalenone	Au@Pt	Multiplexed lateral flow immunoassay	0.24 and 0.04 ng/mL	Corn, wheat and water	[125]
Aflatoxin B1	AuPt@CeO_2_	Electrochemical	2.13 fg/mL	Walnuts, oats, Quisqualis Fructu and Radix astragali	[130]
Aflatoxin B1	AuPtNPs/Ni–Co NCs	Colorimetric and electrochemical dual-mode	0.49 and 0.76 pg/mL	Edible oil, peanut, and cornmeal	[139]
**Heavy metal ions**					
Hg^2+^, Pb^2+^, Co^2+^, Cr^6+^, and Fe^3+^	AuPt@Fe-N-C, AuPt@N-C, and Fe-N-C	Colorimetric sensor array	0.5 μM	Seawater and salmon	[114]
**Antibiotic, pesticide and veterinary drug residues**					
Parathion	Au@Pt	Colorimetric (Bio-barcode)	2.13 × 10^–3^ μg/kg	Rice, pear, apple, and cabbage	[41]
Malathion	CeO_2_ nanorods@AuPt	Colorimetric	1.5 nM	Cucumber juice and human serum	[54]
Carbendazim	Au@Pt	Colorimetric	0.038 ng/mg	Leeks and rice	[46]
Glutathione	Capreomycin@AuPt	Colorimetric	0.58 μM	Tomato supernatant	[52]
Acid phosphatase and malathion	Au@Pt porous nanospheres	Colorimetric	0.047 U/L and 1.96 nM	Fetal bovine serum and cucumber juice	[56]
Enrofloxacin	Au@Pt nanoflowers/CoSe_2_	Electrochemical	1.59 fg/mL	Milk	[61]
3-Phenoxybenzoic acid	Au@Pt	Lateral flow immunoassay	0.005 ng/mL	Milk and lake water	[63]
Gentamicin, streptomycin, and clenbuterol	Fe_3_O_4_/Au@Pt	Lateral flow immunoassay	10.1, 6.3, and 1.1 pg/mL	Pork, milk, and honey	[73]
Furosemide	Au/Pt@CeO_2_ and Au/Pt@Cu–MOF	Electrochemical	1 ng/L	Diet tea, diet bread and diet capsule	[78]
Ractopamine, clenbuterol, and gentamicin	GO/Au–AuPt	Lateral flow immunoassay	0.013, 0.12, and 0.12 ng/mL	Pork, chicken, lake water, and river water	[83]
Dipterex	Ti_3_C_2_T_x_ MXene@AuPt	Colorimetric	0.479 ng/mL	Insecticide samples	[86]
Tetracycline	Au@Pt/carbon nanotubes	Colorimetric	0.74 ng/mL	Milk and pork	[89]
Ofloxacin	Au@Pt	Lateral flow immunoassay	0.017 ng/mL	Chicken and fish	[97]
Lincomycin	SnS_2_ QDs/Cys-AuPt heterogeneous nanorings	Electrochemiluminescence	0.7 fg/mL	Milk	[111]
Omethoate	Pt@Au	Colorimetric	0.01 μg/L	Chinese cabbage	[120]
Chloramphenicol	Au@Pt	Colorimetric and SERS dual-mode	9.23 × 10^−9^ and 4.96 × 10^−13^ M	Milk	[129]
Acetamiprid	Au@Pt	Lateral flow assay (aptamer)	0.17 ng/mL	Tomato	[143]
Acetamiprid	Au@Pt NPs	Lateral flow assay (colorimetric and catalytic colorimetric)	0.33 ng/mL and 0.068 ng/mL	Tomato and rape	[144]
Imidacloprid	Au@Pt	Colorimetric and fluorescence dual-mode	0.88 and 1.14 μg/L	Cabbage, cucumber, and zucchini	[148]
**Other food hazards**					
Antioxidants	Au_2_Pt	Three-channel colorimetric sensor array	<0.2 μM	Milk, green tea and orange juice	[28]
Okadaic acid	Au@Pt	Lateral flow immunoassay	0.5 ng/mL	Seawater, river water, and fish	[40]
H_2_O_2_	SiO_2_@Au@Pt	Colorimetric	1.0 mM	––	[47]
Myoglobin	Au@Pt	Lateral flow immunoassay	0.15 ng/mL	Beef, chicken, and turkey meat	[53]
Toxin B in *Clostridium difficile*	Au/Pt	Lateral flow immunoassay	1 ng/mL	––	[62]
Saxitoxin	Fe_3_O_4_@Au-Pt	Fluorescence	0.6 nM	Shellfish	[70]
Saxitoxin	Fe_3_O_4_@Au-Pt/MIP	Colorimetric and SERS	3.1 and 0.03 nM	Mussel and clam	[71]
H_2_O_2_	AuPtRu/ZnO-rGO	Colorimetric	3.0 μM	Milk	[84]
Cysteine	MoS_2_-Au@Pt	Colorimetric	0.7 μM	Tablets	[87]
Bisphenol A	Chitosan/MWCNTs-AuPtPd	Electrochemical	1.4 nM	Tap water, orange juice and milk	[88]
Seven phenolic acids	Au@Pt, Au@Os, and Au@Pd	Colorimetric sensor array	0.0032, 0.0017, 0.0031, 0.003, 0.0015, 0.0028, and 0.0013 mM	Tap water, plant, fruit, and Chinese medicine	[123]
H_2_O_2_	AuPt	Electrochemical	2.5 µM	Raw cow milk	[135]
Cysteine	Au@Pt	Colorimetric	1.5 nM	Fetal bovine serum and fresh milk	[142]
L-histidine	Au@Pt	Fluorescence	6.2 μM	Infant formula	[149]
Histamine	Au@Pt@Au	Colorimetric (MIP-assisted ELISA)	0.069 mg/L	Yellow rice wine and liqueur	[150]

Abbreviation: ELISA, enzyme-linked immunosorbent assay; IL, ionic liquids; MIP, molecular imprinted polymer; MWCNTs, multi-walled carbon nanotubes; GO, graphene oxide; QDs, quantum dots; SERS, surface enhanced Raman spectroscopy; ZIF-67, zeolitic imidazolate framework-67.

## Data Availability

No new data were created or analyzed in this study.
